# Diagnosing recurring explanatory gaps and proposing a mechanism-based psychoeducational framework in Islamic-tradition long-term care: a model informed by the moral exemplarity of the prophet Muhammad

**DOI:** 10.3389/fpubh.2026.1950225

**Published:** 2026-09-28

**Authors:** Yaşar Emrah Koşdaş, Mine Çeliköz

**Affiliations:** 1 Department of Islamic History and Arts, Faculty of Theology, Recep Tayyip Erdoğan University, Rize, Türkiye; 2 Department of Lifelong Learning and Adult Education, Faculty of Education, Yıldız Technical University, Istanbul, Türkiye

**Keywords:** Islamic intervention, long-term care, mechanism-based design, modular psychoeducation, Sīra, uswah ḥasanah, vulnerability in later life

## Abstract

This study examines Islamic-tradition psychosocial, religious, and spiritual intervention programs implemented in institutional long-term care settings. As a Hypothesis and Theory contribution, the study adopts a conceptual-theoretical research design. Eight intervention programs identified through a structured search across multiple databases were examined across four analytical dimensions. These dimensions were program theory articulation, mechanism specification, construct-measurement alignment, and methodological integrity. The analysis identified three recurring methodological limitations. These are insufficient specification of component-mechanism-outcome chains; methodological challenges related to fidelity monitoring, control condition design, and role differentiation; and reliance on single-source self-report data without systematic cognitive-capacity screening. The analysis also identified a content-level gap. The outcome architectures of the reviewed programs are organized around constructs such as happiness, depression, and quality of life, while loneliness, self-esteem, and death anxiety are not designated as primary outcomes. These three constructs are among the psychosocial vulnerabilities most prevalent in this population. This pattern points to a gap in the collective target architecture of this literature. The findings suggest that the main issue within the literature examined here is not the absence of intervention effects. Rather, it is the insufficient explanatory infrastructure needed to account for those effects. In response, the study proposes a mechanism-based and modular psychoeducational framework grounded in the uswah ḥasanah tradition. The framework positions the moral exemplarity of the Prophet Muhammad as its pedagogical organizing principle. Through three sequential modules, loneliness, self-esteem, and death anxiety are addressed as primary outcome constructs. Each outcome is structured through explicitly specified component-mechanism-outcome pathways. The framework also incorporates a four-level CIPP (Context-Input-Process-Product) evaluation architecture and a multi-source fidelity-monitoring infrastructure. This structure aims to strengthen implementation transparency and theory testing. By shifting attention from outcome reporting to mechanism specification, the proposed framework advances the conceptual foundations of faith-based psychosocial intervention research in long-term care settings. The model also provides a theory-informed basis for the development, evaluation, and continued refinement of spiritually integrated interventions aimed at promoting psychosocial wellbeing in later life.

## 1 Introduction

The global expansion of the ageing population is intensifying demand for institutional long-term care (LTC) settings. Individuals residing in these contexts require not only physical care but also confront substantial psychosocial and existential challenges (1). In response, a heterogeneous range of intervention programs has proliferated, encompassing secular models, religious and spiritual interventions, meaning-centered therapies, and reminiscence-based approaches. Islamic-tradition programs constitute a distinct and increasingly documented strand within this landscape.

Despite their documented contributions to psychological wellbeing, Islamic-tradition LTC interventions exhibit a recurring explanatory limitation: outcomes are reported, yet the processes through which those outcomes are produced are rarely specified with sufficient clarity. This limitation operates at two interconnected levels. Methodologically, component–outcome relationships remain largely implicit, mechanisms of change are insufficiently articulated, self-report measures predominate, and implementation fidelity is rarely monitored systematically. At the content level, a complementary examination of intervention targets revealed that the psychosocial vulnerabilities most prevalent in institutional LTC settings (loneliness, diminished self-esteem, and death anxiety) are seldom designated as primary outcomes.

The triad was not derived from a single theory. It was defined through three selection criteria applied jointly: documented prevalence in institutional LTC (2–5); the availability of a validated primary outcome instrument (6–8); and non-designation as a primary outcome across the reviewed programs (Section 3.2.1). Each instrument has documented use in samples of the kind this framework addresses: the Rosenberg scale distinguishes nursing-home from community-dwelling respondents (5), the Templer scale has been administered across nursing-home populations (2, 9, 10), and the UCLA scale has been validated for use with older adults and applied in a nursing-home trial within this literature (11, 12); validated adaptations of all three are available for the target contexts (Section 4.2.2). Taken together the three constructs correspond to the relational, self-structural, and existential dimensions of later-life vulnerability (13), the developmental scheme on which the framework’s mechanism logic is built (Section 4.2.1). A further constraint follows from the model’s architecture rather than from these criteria. Meaning loss, for example, is well documented among nursing-home residents (14, 15), but meaning-focused reappraisal and ego-integrity facilitation are the specified mechanisms of Modules 1 and 3; designating meaning loss as a primary outcome would place the same construct at both ends of those pathways, so that the mediator would covary with the outcome by definition rather than by intervention and Proposition P1 could not be tested against it [(16); Table 1]. The reviewed programs designate other outcomes as primary (depression, quality of life, happiness, and general mental health), and these remain prominent concerns in this population (17, 18). The triad is therefore an empirically informed and deliberately bounded specification, not an exhaustive account of psychosocial vulnerability in institutional LTC; it adds targets to the field’s outcome architecture rather than replacing what that architecture already measures.

**Table 1:** Module–mechanism–outcome overview.

Module	Primary target	Core mechanism	Outcome indicator
Module 1	Loneliness	Meaning-focused reappraisal; behavioral reinforcement	UCLA Loneliness Scale
Module 2	Self-esteem	Generativity reactivation; relational constitution of self-worth; self-compassion activation	Rosenberg Self-Esteem Scale
Module 3	Death anxiety	Cognitive reappraisal; ego integrity facilitation; structured mortality exposure	Templer Death Anxiety Scale

CMO, component–mechanism–outcome.

Addressing these limitations requires what the present study terms explanatory infrastructure. This infrastructure comprises three interrelated elements. The first is the explicit specification of component–mechanism–outcome pathways. The second is the establishment of multi-source data structures that allow independent verification of intervention effects. The third is the direct operationalization of core psychosocial vulnerabilities as primary outcome constructs. The distinction between specified and unspecified mechanisms is consequential. Explicitly specified mechanisms generate testable and potentially falsifiable propositions that can be examined empirically, whereas unspecified mechanisms limit explanatory development regardless of the magnitude of observed effects (16, 19).

This study aims to examine religious and spiritual intervention programs for older adults in LTC settings, focusing on their methodological and content-related characteristics, and to propose a mechanism-based, modular psychoeducational framework grounded in the moral exemplarity of the Prophet Muhammad to address the identified limitations. This is a conceptual, theory-building contribution: it develops and specifies a framework whose component–mechanism–outcome pathways are advanced as testable propositions rather than as empirically validated outcomes.

To address the study aim, the following analytical and framework development research questions were formulated.

### 1.1 Analytical research questions

*RQ1*. How are existing Islamic-tradition religious and spiritual intervention programs implemented in long-term care settings structured in terms of program logic, mechanism specification, and methodological integrity?

*RQ2*. How, and to what extent, do these programs directly target the core psychosocial vulnerabilities of loneliness, self-esteem, and death anxiety as primary outcomes?

### 1.2 Framework development research question

*RQ3*. How can the methodological and psychosocial gaps identified through this analysis be addressed through a mechanism-based and modular psychoeducational framework grounded in the moral exemplarity of the Prophet Muhammad?

The framework’s pedagogical content is grounded in the moral exemplarity of the Prophet Muhammad. The Qurʾānic designation of the Prophet as “a most excellent exemplar” (Qurʾān 33:21), together with the injunction to follow his guidance (Qurʾān 4:65), establishes his example as both a theological principle and an educational resource. Within the Islamic intellectual tradition, this exemplarity is theorized through the concept of uswah ḥasanah (“the beautiful model”), a normative ethical framework for character formation (taʿlīm wa-tarbiya) across the lifespan (20–22). The Hadith Tarbawi tradition systematizes it further into cognitive, demonstrative, affective, and evaluative dimensions of learning (23). The use of Sīra-based content thus derives from an established Islamic educational tradition rather than from a novel theoretical claim; the Prophet’s moral authority has, moreover, been recognized across diverse intellectual traditions (24–26).

Beyond its theological legitimacy, the life of the Prophet Muhammad contains themes that correspond closely to the three psychosocial vulnerabilities targeted in the present framework. His inclusion of older individuals in social and decision-making processes [(27), Adāb 66; (28)] reflects patterns of relational belonging and social recognition directly relevant to interventions addressing loneliness. His retreat to the Cave of Ḥirāʾ [(29), Badʾ al-Waḥy] offers a culturally authoritative instance in which being alone is not identical with being diminished. The episode is engaged here as a contrastive resource for the reappraisal of solitude rather than as an analogue of institutional aloneness, whose involuntary and open-ended character the framework treats as materially different (Section 4.2.2). So understood, the reading is consistent with Moustakas’ (30) account of constructive solitude and with Frankl’s (31) account of meaning. The Prophet’s consistent positioning of older individuals as respected contributors whose accumulated knowledge and experience retain social value (32) enacts the generative orientation that Erikson (13) identifies as the primary developmental source of self-worth in later life, the disruption of which McAdams and de St. Aubin (33) directly link to self-worth erosion. This thereby provides a Sīra-based resource for addressing diminished self-esteem in institutional LTC settings. Likewise, the Prophet’s encouragement of death remembrance and his composed orientation toward mortality [(34), Zuhd, 3] bear conceptual similarities to contemporary approaches emphasizing acceptance, meaning-making, and spiritual coping in response to death anxiety (35, 36). Together they reframe death not as a threat to be avoided but as an existential reality to be understood and integrated, a reorientation consistent with Heidegger’s (37) account of authentic being-toward-death.

Empirical and theoretical literature supports this adaptation; the evidence is set out where the mechanisms are specified (Section 4.2.1). What has not been undertaken in the existing literature is the systematic operationalization of Sīra-based content within an institutional LTC intervention explicitly targeting loneliness, self-esteem, and death anxiety. Taken together, these considerations provide a coherent foundation for an integrated intervention framework centered on the three core vulnerability domains identified in this study.

## 2 Methods

This study adopts a conceptual–theoretical research design that prioritizes explanatory clarity over empirical findings. In line with Jaakkola’s (38) typology of conceptual research designs which distinguishes among theory synthesis, model building, typology development, and conceptual framework construction, the present study combines theory synthesis and model building. Theory synthesis identifies structural patterns across existing programs; model building translates those patterns into a corrective framework, thereby addressing the study’s dual objective: diagnosing recurring design limitations and deriving a mechanism-based response. The analysis does not aim to aggregate outcomes across interventions. Instead, it adopts a theory-driven, diagnostic approach to examine program logic and trace recurring design choices.

The literature search was conducted across Scopus, Web of Science, PubMed, and the National Thesis Center of the Council of Higher Education (Türkiye). Keywords including loneliness, self-esteem, death anxiety, spiritual care, religious intervention, psychoeducational program, older adults, and long-term care were used in multiple combinations. The search was restricted to peer-reviewed journal articles and graduate theses published between 2015 and 2025. Because intervention research in this field is published partly in regionally indexed journals that the major international databases do not cover, these queries were supplemented by backward reference screening and forward citation tracking. Several of the retrieved programs, including work published in Iranian and Indonesian outlets, entered the corpus through this route rather than through direct database hits. Searches were completed in early 2026 and yielded 112 records; following title and abstract screening and subsequent full-text assessment, eight studies met all inclusion criteria, and tracking the reference lists and the citing articles of those eight returned no further eligible record. Even so, no search of this kind can be shown to be exhaustive: work indexed by none of these sources and cited by none of the included programs would lie outside the reach of both procedures and may therefore remain unretrieved. The corpus is accordingly treated as the retrievable literature rather than a demonstrably exhaustive one, and the counts reported below characterize this corpus rather than the literature as a whole.

Studies were included in the core analytical sample when they met all four of the following criteria: (i) conducted in institutional long-term care (LTC) settings; (ii) involving a structured intervention with clearly defined components; (iii) incorporating an explicitly identifiable Islamic religious or spiritual component; and (iv) providing sufficient methodological and conceptual detail to enable analysis of program structure and implementation processes. Following title and abstract screening, studies that clearly failed to meet one or more of these criteria were excluded. The remaining studies were assessed at the full-text level; studies were additionally excluded when methodological reporting was insufficient to reconstruct the intervention’s structural characteristics or assess its implementation processes. This procedure resulted in the inclusion of eight studies in the core analytical sample.

### 2.1 Core analytical sample (*n* = 8)

- Heidari et al. (39)—Iran: A 90-day structured spiritual care program implemented in a nursing home in Isfahan, consisting of prayer, Qurʼān recitation, and spiritual counseling components.
- Ayyari et al. (40)—Iran: Four-week group sessions conducted in Sabzevar with an all-female sample (*n* = 38), focusing on active listening, the support of religious rituals, and the cultivation of hope.
- Setiowati et al. (41)—Indonesia: A psycho-religious group therapy program implemented in a nursing home, consisting of dhikr practices and group sharing sessions (*n* = 20).
- Dağcı (42)—Türkiye: A 12-session value-oriented spiritual care program developed for older adults residing in nursing homes (*n* = 38) based on a qualitative needs analysis and addressing themes such as forgiveness, gratitude, and trust in God (tawakkul).
- Pramesona and Taneepanichskul (43)—Indonesia: A 12-week quasi-experimental study conducted in three nursing homes in Yogyakarta (*n* = 60), combining Qurʼānic recitation sessions with sermon-based interventions targeting depressive symptoms and quality of life.
- Minarti et al. (44)—Indonesia: An experimental pre-test post-test study conducted in two nursing homes in Surabaya (*n* = 30), delivering a combination of counseling and dhikr interventions over 8 weeks, targeting self-acceptance.
- Rumakey et al. (45)—Indonesia: A quasi-experimental study examining the effects of a combined spiritual–cognitive therapy on depression among older adults in nursing homes (*n* = 30).
- Ramazani and Bakhtiari (12)—Iran: A quasi-experimental intervention consisting of structured spiritual therapy sessions targeting loneliness among older adults in a nursing home setting (*n* = 15).

Each intervention program was examined across four analytical dimensions: program theory articulation, mechanism specification, construct–measurement alignment, and methodological integrity, encompassing implementation fidelity, control condition design, and data source diversity. The analysis evaluated the extent to which intervention components were embedded within an explicit component–mechanism–outcome logic and linked to corresponding outcome measures. Cases in which no explicit component–mechanism–outcome chain could be identified were coded as indicative of limited program theory articulation or insufficient mechanism specification.

Since no single theoretical framework fully encompasses all four analytical dimensions, the integrated use of multiple theoretical frameworks was necessary (The correspondence between each dimension and the framework applied to it is set out in Table 2). The extent to which intervention components were explicitly linked to mechanisms and outcomes was assessed using two frameworks. The MRC framework (19, 46) requires explicit articulation of how components produce outcomes. Realist evaluation (47) requires that mechanisms be specified together with the contexts that activate them (context–mechanism–outcome configurations). Although the MRC framework originated in clinical intervention research, its 2021 update explicitly extends its scope to behavioral, social, and psychoeducational interventions (46, p. 2); its application here is therefore methodologically appropriate for the program types under review. Internal validity was evaluated using the Cochrane Risk of Bias framework (48), focusing on role overlap, lack of blinding, and other sources of bias. Intervention fidelity literature was used to assess whether implementation was delivered consistently across participants (49). Evidence on the Hawthorne effect was applied when evaluating passive control groups and attention-related influences (50). Control condition design literature was used to assess whether studies were able to isolate intervention-specific effects (51). At the measurement level, studies relying exclusively on self-report data without independent validation were evaluated within the framework of construct validity theory (52). Common-method variance and age-related self-report considerations were additionally evaluated with reference to Podsakoff et al. (53) and Schwarz and Knäuper (54). The adequacy of explanations regarding how intervention effects are produced was evaluated based on the mechanisms of change literature (16), which shows that mechanisms often remain unspecified even in well-established interventions, situating the observed mechanism ambiguity as a structural issue rather than a study-specific limitation. These Western-derived frameworks are applied here evaluatively rather than normatively: they assess the explanatory adequacy of existing programs through mechanism articulation, fidelity, control design, and data-source diversity not the legitimacy of Islamic-tradition content.

**Table 2:** Analytical framework applied across all eight studies.

Analytical dimension	Evaluative focus	Framework applied
Program theory articulation (→ 3.1.1)	Explicit articulation of component–mechanism–outcome logic	MRC framework (19, 46); Realist Evaluation (47)
Mechanism specification (→ 3.1.1)	Whether the psychosocial change process is named and tied to a defined component	Mechanisms-of-change literature (16); Realist Evaluation (47)
Construct–measurement alignment (→ 3.2.1)	Whether loneliness, self-esteem, or death anxiety is designated as a primary outcome and measured with a validated instrument	Construct validity theory (52); LTC psychosocial vulnerability literature (2, 4, 5)
Methodological integrity (→ 3.1.2, 3.1.3)	Fidelity monitoring; control condition design; role separation; data-source independence and cognitive capacity screening	Cochrane RoB (48); Bellg et al. (49); Mohr et al. (51); McCarney et al. (50); Podsakoff et al. (53); Schwarz and Knäuper (54)
Arrows indicate the Findings subsection in which each dimension is reported; program theory articulation and mechanism specification are reported jointly in 3.1.1. Each dimension was established as a stable reference prior to study appraisal.		

Distribution of the four analytical problem areas across reviewed programs (Summary matrix)						
Study	3.1.1 Program theory	3.1.2 Methodological integrity			3.1.3 Data sources	3.2.1 Construct targeting
	Unspecified CMO	Absent Fidelity	Passive Control	Role Conflation	Single Source	Core Constructs Not Designated
Heidari et al. (39)	✓	✓	✓	✓	✓	✓
Ayyari et al. (40)	✓	✓	✓	✓	✓	✓
Pramesona and Taneepanichskul (43)	✓	✓	✓	✓	~	✓
Setiowati et al. (41)	✓	✓	✓	✓	✓	✓
Rumakey et al. (45)	✓	✓	✓	–	~	✓
Ramazani and Bakhtiari (12)	✓	✓	✓	–	✓	~
Minarti et al. (44)	✓	✓	✓	–	✓	~
Dağcı (42)	~	~	✓	–	~	✓
✓ = feature present in the reported procedures; for the final column, none of the three constructs is designated as a primary outcome. ~ = partially present or mitigated; in the data-source column this covers two distinct cases, a multi-source data architecture in Dağcı (42), and additional instruments serving screening or background purposes rather than independent outcome measurement in Pramesona and Taneepanichskul (43) and Rumakey et al. (45). – = not determinable from the reported procedures; no inference is drawn about the underlying design.						

Characterization of the four analytical problem areas			
	Limitation	Core problem	Studies affected
3.1.1	Implicit/under-specified program theory	Components described; mechanisms implicit; no testable CMO chains across all 8 programs, partially mitigated in 1 (42).	All 8 studies (varying severity; (42) partially mitigated)
3.1.2	Absent implementation fidelity	Delivery, receipt, and enactment unmonitored; effects not design-attributable.	Heidari et al. (39); Ayyari et al. (40); Pramesona and Taneepanichskul (43); Setiowati et al. (41); Rumakey et al. (45); Ramazani and Bakhtiari (12); Minarti et al. (44)
3.1.2	Passive control conditions	Control groups receive no attention-matched equivalent. Non-specific (Hawthorne) effects cannot be ruled out.	Heidari et al. (39); Ayyari et al. (40); Pramesona and Taneepanichskul (43); Setiowati et al. (41); Rumakey et al. (45); Ramazani and Bakhtiari (12); Minarti et al. (44); Dağcı (42)
3.1.2	Role conflation and detection bias	Same individual delivers intervention and collects outcome data. Performance and detection bias per Cochrane RoB framework.	Heidari et al. (39); Ayyari et al. (40); Pramesona and Taneepanichskul (43); Setiowati et al. (41)
3.1.3	Reliance on single-source self-report data	No independent data source corroboration. Cognitive capacity not screened in 6 of 8 studies. Self-report validity compromised in LTC populations.	Heidari et al. (39); Ayyari et al. (40); Setiowati et al. (41); Ramazani and Bakhtiari (12); Minarti et al. (44)—partially: Pramesona and Taneepanichskul (43); Rumakey et al. (45)
3.2.1	Core psychosocial vulnerabilities as non-designated outcomes	Loneliness, self-esteem, and death anxiety are not designated as primary outcomes; the primary outcome position is occupied by proximal but non-equivalent constructs (happiness, QoL, depression).	No program designates more than one of the three. Loneliness is designated in 1 of 8 (12); self-esteem in none, approached through the adjacent construct of self-acceptance in 1 of 8 (44); death anxiety in none.

Core psychosocial vulnerabilities: theoretical basis, LTC prevalence, and targeting status					
Loneliness	Perlman and Peplau (61); Weiss (62); Erikson (13)	~50.7% institutionalized; 61% moderate, 35% severe loneliness in NHs	Salari et al. (4); Gardiner et al. (3); Lapane et al. (95)	1 of 8 studies only	Ramazani and Bakhtiari (12)—UCLA Loneliness Scale
Diminished Self-Esteem	Rosenberg (6); Harter (63); Erikson (13)	RSES median 37.0 (NH) vs. 40.0 (community), *p* = 0.002	Šare et al. (5)	None	None—Minarti et al. (44) uses self-acceptance (non-equivalent)
Death Anxiety	Yalom (36); Becker (64); Wong (65); Erikson (13)	DAS mean: 6.10–8.39 across NH populations	Ergin et al. (2); Akçay and Kurt Magrebi (9); Yousefi Afrashteh et al. (10).	None	None
DAS, Death Anxiety Scale; RSES, Rosenberg Self-Esteem Scale; NH, nursing home; QoL, quality of life; UCLA, University of California Los Angeles Loneliness Scale.					

The analytical framework was systematically applied across all reviewed studies. Independently, a complementary content-level evaluation compared the outcomes targeted in each program against the three psychosocial vulnerabilities most empirically prevalent in institutional LTC populations: loneliness, diminished self-esteem, and death anxiety (2, 4, 5).

Each of the four analytical dimensions was operationalized through an explicit three-level coding scheme (present/partial/absent) applied uniformly across all eight programs prior to interpretive synthesis. Program theory articulation was coded present when an explicit component–mechanism–outcome statement was identifiable, partial when components and outcomes were linked without a specified mechanism, and absent when no causal linkage was articulated. Mechanism specification was coded present when the psychosocial process assumed to be activated was named and tied to a defined component, partial when a process was named without component linkage, and absent when no change process was stated. Construct–measurement alignment was coded present when at least one of the three core vulnerabilities (loneliness, self-esteem, death anxiety) was designated as a primary outcome and measured with a validated instrument, partial when a conceptually adjacent construct was targeted, and absent when none of the three was addressed. Methodological integrity was coded across four binary sub-criteria, fidelity monitoring, attention-matched control, role separation, and data-source independence, with a study classified as present only when all four were satisfied, partial when at least one was satisfied, and absent when none was.

The scheme was applied independently by the first and second authors, working at separate institutions, to all eight programs, yielding 32 study-by-dimension coding decisions per coder. Before any comparison of the two codings and before any consensus discussion, the same code was assigned in 27 of the 32 decisions (84.4%), giving Cohen’s *κ* = 0.57 (95% CI [0.24, 0.90]). Both raw and chance-corrected figures are given because the marginal distribution is strongly skewed: no decision was assigned to the highest category by either coder, and 23 and 26, respectively, fell in the lowest, so that the effective scale was binary and a linearly weighted coefficient is identical to the unweighted one. This distribution reflects the uniformity of the pattern documented in Section 3.1 rather than any property of the coders; under such marginals chance agreement is high (Pₑ = 0.64) and κ is correspondingly conservative relative to observed agreement, a divergence documented as a known property of the coefficient (55). Neither figure is offered as a population-level reliability estimate; both describe consistency across a small, fully enumerated decision set.

All five divergences fell at the partial/absent boundary and arose in four studies (12, 42, 44, 45): two in program theory articulation, two in mechanism specification, and one in methodological integrity. Four concerned whether a study’s reporting met the threshold for partial articulation or for a named change process; the fifth arose where two of the four methodological-integrity sub-criteria could not be determined from the study’s report. They were resolved by returning to the source texts and to the thresholds fixed in advance, independently of the summary judgments subsequently tabulated. The rule applied was that silence in the reported procedures is treated as not determinable rather than as evidence of absence, so that no inference is drawn about the underlying design, and that rule is reflected in the notation of Table 2. Consensus was reached on all five, and the full decision-level record is available from the corresponding author. The safeguard against confirmatory bias therefore rests on three features: the four dimensions and their three-level thresholds were fixed before study appraisal began; the first coding stage was conducted independently and without contact; and the resolution rule was applied uniformly rather than case by case. The resulting study-level judgments were tabulated and presented in the Findings section. Table 3 summarizes the five-stage analytical procedure used in this study.

**Table 3:** Analytical logic of the study: from literature identification to framework derivation.

Stage	Analytical procedure		Frameworks and specifications
1	Research design Conceptual–theoretical study aimed at enhancing explanatory clarity. Theory synthesis employed to identify structural patterns; model building adopted to derive a corrective framework.		Jaakkola (38): Theory synthesis + model building Design type: Diagnostic/explanatory Orientation: Non-empirical, pattern-driven
2	Literature and content search Structured search across four databases, supplemented by backward reference screening and forward citation tracking because part of this literature appears in regionally indexed journals not covered by the major international databases. Keywords: loneliness, older adults, self-esteem, death anxiety, spiritual care, religious intervention, psychoeducational program, long-term care.		Sources: Scopus, Web of Science, PubMed, National Thesis Center (Türkiye) Period: 2015–2025, Searches completed: early 2026 Records retrieved: 112 → final analytical sample: 8 studies Corpus status: retrievable literature; exhaustiveness not demonstrable. Pedagogical content: al-Kutub al-Sitta accessed through thematic chapter headings (Adāb, Birr, Badʾ al-Waḥy, Zuhd, Janāʾiz, Wasāyā); primary sīra, ṭabaqāt and tārīkh sources; later devotional and homiletic compilations excluded.
3	Sample and content selection Core analytical sample (*n* = 8): institutional LTC settings with sufficient conceptual and methodological detail for CMO analysis.		Inclusion criteria: institutional LTC, structured intervention, Islamic dimension, sufficient methodological detail. Content criteria: theme carried by more than one independent report or episode, authenticity grading checked, no contextual distortion or doctrinal overreach.
4	Analytical procedure Each program examined across four dimensions: program theory articulation, mechanism specification, construct–measurement alignment, methodological integrity. Frameworks established as stable reference prior to data engagement. Independent coding by two researchers at separate institutions; pre-consensus agreement 84.4% (27/32), *κ* = 0.57; consensus-based resolution of five divergences.		Program theory: MRC ((19); (46)), Realist Evaluation (47). Mechanism specification: Kazdin (16). Construct–measurement alignment: Messick (52). Methodological integrity: Cochrane RoB (48), Bellg et al. (49), Mohr et al. (51), McCarney et al. (50), Podsakoff et al. (53), Schwarz and Knäuper (54), Inter-coder agreement: kappa paradox under skewed marginals (55).
Analytical output			
	Analytical findings Four recurrent problem areas identified through iterative framework application: implicit program theory; methodological integrity cluster; single-source data and absent cognitive screening; non-designation of the core constructs as primary outcomes. A1 Implicit program theory A2 Methodological integrity cluster A3 Single-source data and absent cognitive screening A4 Core constructs not designated as primary outcomes	→	Framework derivation Each design component developed as a direct response to one or more identified problem areas.

MRC, Medical Research Council; RoB, Risk of Bias; CMO, component–mechanism–outcome; LTC, long-term care. Here CMO denotes component–mechanism–outcome in the framework sense; in realist evaluation CMO denotes context–mechanism–outcome configurations.

The prophetic material used in the three modules was selected through a specified procedure whose orientation was deductive at the level of the search frame and inductive at the level of theme construction. Three search domains were fixed in advance from the vulnerability triad: relational belonging and the experience of solitude, worth constituted independently of functional capacity, and the remembrance of death. Retrieval within the canonical hadith collections proceeded through the thematic chapter headings (abwāb) corresponding to these domains, principally Adāb, Birr and Badʾ al-Waḥy for the first, Adāb for the second, and Zuhd, Janāʾiz and Wasāyā for the third. It was extended to the primary sīra, ṭabaqāt and tārīkh sources cited in this article (56–58), in which the material bearing on the second domain principally lies; later devotional and homiletic compilations were excluded. Reports and episodes were coded for the psychosocial situation they depict rather than for their juridical content, following established procedures for the thematic coding of qualitative source material (59), and a theme was retained only where carried by more than one independent report or episode. Retained reports were checked against the authenticity grading of the source collection, and episodes were removed where the intended reading exceeded what the narrative context supports or assigned a theological function beyond the report’s evidentiary weight. The record of this selection is available from the corresponding author.

The pedagogical content of the three modules was additionally submitted in draft form to seven specialists in curriculum and adult education, psychology of religion, religious education, and Islamic history, each consulted individually rather than as a panel. They were asked to comment on the fit between the selected prophetic material and the mechanism each module specifies, and on its suitability for an older institutional audience; the content was revised where a mismatch was identified. This was an author-initiated consultation carried out during drafting: no rating scheme was applied, no agreement threshold was set, and the responses were not aggregated. It is therefore distinct in timing and in function from the independent appraisal specified in Section 4.2.3, which is prospective, excludes the authors, and rates the framework on the same four-dimension scheme applied to the reviewed corpus.

## 3 Findings

### 3.1 Methodological limitations

The systematic application of the analytical framework to the eight programs revealed that recurring methodological gaps converged around three problem areas, addressed in turn below (Table 2).

#### 3.1.1 Implicit program theory and under-specified mechanism pathways

The reviewed studies specify intervention components and report outcomes, but rarely name the psychosocial process assumed to link them; where such a process is named, it is not tied to a defined component, and the contextual conditions under which it is expected to operate are left unspecified. Component–mechanism–outcome relationships thus remain largely implicit rather than articulated as explicit, testable causal models, leaving mechanism attribution indeterminate at the causal level.

This pattern is most clearly evident in Minarti et al. (44): counseling and dhikr are combined to promote self-acceptance and psychological comfort, and positive outcomes are reported, yet whether the observed effects result from cognitive reframing, emotional regulation, spiritual meaning-making, or some interaction among these processes remains unspecified. Pramesona and Taneepanichskul (43) present a similar case: Qurʾānic recitation and sermon-based instruction are delivered together, improvements in depression and quality of life are reported, and the authors themselves acknowledge that the effects of these components cannot be separated. The same limitation recurs in Rumakey et al. (45), where detailed procedural description leaves unexplained whether reductions in depressive symptoms are attributable to cognitive processes, spiritual engagement, or their interaction, and in Ramazani and Bakhtiari (12), where elements such as responsibility-taking, reliance on God, and meaning-making are not systematically linked to defined mechanisms of change. The core limitation across these cases is not the absence of an intervention rationale. Rather, it is the absence of mechanism specification sufficient to render observed effects analytically interpretable, empirically testable, and replicable across contexts. The pattern is present across all eight programs, partially mitigated only in Dağcı (42) (Table 2).

#### 3.1.2 Absent fidelity, passive controls & role conflation

Implementation fidelity, control condition design, and role separation were examined together for each program as an integrated design structure; their distribution across the eight studies is summarized in Table 2.

In Heidari et al. (39), all three design features remain unaddressed in the reported procedures. The same researcher delivered the intervention and conducted outcome assessments; the control group was passive, without provisions for balancing attention, therapeutic contact, or expectancy; and no systematic fidelity monitoring is reported. Pramesona and Taneepanichskul (43) present the same configuration: the control condition does not isolate intervention-specific effects, fidelity is neither standardized nor monitored across delivery, receipt, and enactment, and blinding and therapist allegiance balancing are unspecified. Ayyari et al. (40) report a formally structured RCT in which fidelity monitoring is nonetheless absent, despite a multi-component design encompassing active listening, facilitation of religious practices, supportive presence, and hope induction. The control group received routine care with no attention-matched equivalent, while the intervention group received sustained relational engagement including daily interaction and emotional support. At the role level, the researcher who delivered the relationally embedded intervention also served as participants’ main point of relational contact during the study, which is an overlap of functions that remains unaddressed even where baseline assessment and statistical analysis are blinded. Setiowati et al. (41) present the same pattern, the researcher having individually accompanied each participant during data collection and explained every scale item, a procedure that placed the researcher in both the intervention and the measurement context. Rumakey et al. (45), Ramazani and Bakhtiari (12), Minarti et al. (44), and Dağcı (42) present structurally equivalent configurations of passive control and unmonitored fidelity, with role separation undescribed; in Ramazani and Bakhtiari (12), follow-up measurement is additionally absent, restricting outcome data to the immediate post-intervention interval (Table 2).

Across the corpus, fidelity monitoring is absent in seven programs and only partially documented in one (42), and none of the reviewed programs reports an attention-matched comparison condition. In four programs, the reported procedures indicate that the same researcher delivered the intervention and took part in outcome measurement (39–41, 43); in the remaining four, role separation is not described in the reported procedures. In two programs (44, 45), the comparison group was additionally drawn from a different institution (Table 2).

#### 3.1.3 Single-source self-report data and absent cognitive capacity screening

Across the reviewed studies, psychosocial outcomes were assessed predominantly through self-report scales administered to participants under uniform response conditions.

Dağcı (42) reports the most differentiated data structure, combining observation, dyadic interviews, and quantitative scales. Pramesona and Taneepanichskul (43) and Rumakey et al. (45) report additional instruments alongside their outcome scales, although in both cases these served screening or background purposes rather than independent outcome measurement. In the remaining five studies, which include Heidari et al. (39), Ayyari et al. (40), Setiowati et al. (41), Ramazani and Bakhtiari (12), and Minarti et al. (44), outcome data derive from a single self-report source. In Minarti et al. (44), the measurement instrument is reported without scale identification or psychometric properties, which limits the interpretability of the reported scores (Table 2). Cognitive capacity was screened with a standardized instrument in one study only: Rumakey et al. (45) applied an MMSE cut-off (>21). In Pramesona and Taneepanichskul (43), cognitive status was ascertained from medical records and staff information rather than through standardized screening. In the remaining studies, cognitive status was addressed, where addressed at all, through general eligibility criteria without a specified instrument, such as the exclusion of “cognitive disorders such as dementia and Alzheimer” in Heidari et al. (39), and inclusion conditional on being “not cognitively impaired” in Minarti et al. (44).

### 3.2 Content-level findings on outcome designation

The complementary content-level evaluation compared the outcome constructs targeted in each program against the three core vulnerabilities specified in the Method; the results are presented below.

#### 3.2.1 Core psychosocial vulnerabilities as non-designated outcomes

This comparison operates at the level of the field’s outcome architecture: it asks which constructs the literature designates as primary outcomes, not how effectively individual programs moved the outcomes they designated. What it identifies is a what-gap in the sense developed in Section 4.1.

None of the eight reviewed programs directly targets loneliness, self-esteem, and death anxiety together as primary outcome variables, and the majority engage none of the three (Table 2). The consistent pattern across the corpus is the designation of proximal constructs as primary outcomes. Heidari et al. (39) assess perceived stress and general mental health, measured via the Perceived Stress Scale and the GHQ-28. Ayyari et al. (40) designate happiness as the primary outcome, measured via the Oxford Happiness Questionnaire administered at baseline, post-intervention, and one-month follow-up. Pramesona and Taneepanichskul (43) designate depressive symptoms as the primary outcome and quality of life as the secondary outcome, measured via the GDS-15 and the WHOQOL-BREF; Setiowati et al. (41) assess quality of life via the WHOQOL-BREF; and Rumakey et al. (45) assess depression via the GDS within a combined spiritual–cognitive approach. Dağcı (42) targets hope, psychological wellbeing, and perception of God, each measured with a corresponding validated scale.

Two programs approach individual elements of the triad. Minarti et al. (44) address self-acceptance through combined counseling and dhikr, measured with a questionnaire whose scale identity and psychometric properties are not reported, rather than with a validated self-esteem instrument. Ramazani and Bakhtiari (12) constitute the sole program in the corpus to designate one of the three constructs, namely loneliness, as a primary outcome, measured with a validated loneliness scale (60); self-esteem and death anxiety are not among their outcome variables.

Across the eight programs, therefore, loneliness appears as a designated primary outcome in one study; self-esteem appears in none, approached only indirectly through the adjacent construct of self-acceptance in one study; and death anxiety appears in none, in either direct or adjacent form. Where content related to the three vulnerabilities occurs, it appears within intervention themes rather than as measured outcome variables (Table 2).

Viewed across the eight programs, the features documented above do not appear in isolation but as a recurrent joint configuration. On the methodological side, the elements are mutually dependent: where program theory leaves mechanisms unnamed, fidelity monitoring has no defined referent; where comparison conditions are passive or undescribed, non-specific effects remain unseparated from intervention content; and where outcome data derive from a single self-report source without standardized cognitive screening, reported change is difficult to corroborate within the reported designs. On the content side, the same programs are consistently organized around religious content and general psychological wellbeing, while loneliness, self-esteem, and death anxiety are not designated as measured outcomes. The two partial exceptions, which include the multi-source data structure of Dağcı (42) and the direct loneliness targeting of Ramazani and Bakhtiari (12), occur in different programs and do not alter this joint configuration. Within this corpus, therefore, the co-occurrence is systematic rather than incidental: methodological under-specification and non-designation of the core constructs appear together across countries, intervention formats, and delivery contexts. The term is used descriptively of the eight programs examined here and is not advanced as a field-level generalization; establishing whether the same configuration characterizes the wider literature would require an exhaustive retrieval this corpus does not provide. What can be said on this evidence is that within these eight programs the limiting factor is not the absence of reported effects but the absence of explanatory infrastructure sufficient to account for them.

## 4 Discussion

### 4.1 Critical evaluation of existing interventions

Taken together, the findings reveal a recurring design profile across the reviewed programs. Regarding methodological structure (RQ1), the reviewed interventions specify what is delivered and what is measured, while the connecting layer, namely named mechanisms, monitored delivery, balanced comparison conditions, and corroborated measurement, remains largely unreported. Regarding construct designation (RQ2), loneliness, diminished self-esteem, and death anxiety are seldom designated as primary outcomes; the primary outcome position is instead occupied by proximal but non-equivalent indices such as happiness, depression, or quality of life. These features recur with sufficient consistency to constitute an indicative shared design profile, advanced as a hypothesis for confirmation in larger samples rather than as an established generalization. The two findings are gaps of different kinds: RQ1 identifies a how-gap, concerning the processes through which reported effects are produced, and RQ2 a what-gap, concerning which constructs the field designates as primary outcomes. Neither is a defect of the individual studies; both are openings that the proposed framework is built to address.

These observations should not be interpreted as a verdict on the quality of individual studies. Several programs reflect careful methodological work conducted within genuine institutional and resource constraints. Dağcı (42) combines observation, dyadic interviews, and quantitative scales within a multi-source data architecture; Rumakey et al. (45) applied a standardized cognitive screen at intake, and Pramesona and Taneepanichskul (43) ascertained cognitive status from medical records; Ramazani and Bakhtiari (12) measured loneliness through a validated instrument, demonstrating that direct construct targeting is feasible within this tradition. The dominant pattern therefore reflects a remediable design orientation rather than an inherent limitation of the Islamic tradition itself. At the same time, none of these studies addresses the full configuration of limitations identified here: the components of a more comprehensive solution exist in the literature, but in fragmented form.

The designation of proximal constructs in place of the three targets is theoretically consequential, because the two sets of constructs are not interchangeable. Loneliness denotes a perceived relational discrepancy distinct from social isolation or depressive affect (61, 62); self-esteem refers to the global evaluation of one’s worth, which wellbeing or self-acceptance measures capture only partially (6, 63); and death anxiety is organized around the perceived threat of non-existence rather than general distress (8, 36, 64, 65). Within Erikson’s (13) framework, the three correspond to the relational, self-structural, and existential dimensions of late-life vulnerability whose disruption institutional care most consistently produces (33). Their prevalence in LTC populations is empirically substantial, with loneliness affecting roughly half of residents, and institutional samples show lower self-esteem and elevated death anxiety relative to community-dwelling peers (2–5, 66). Measuring happiness or quality of life therefore cannot substitute for measuring these constructs directly.

What unifies these observations is a design convention rather than a belief attributable to any research team. Within this corpus, the question of whether religious or spiritual content is itself sufficient to generate psychosocial change has not had to be posed, because no design was constructed to test it; the premise therefore remains implicit in the designs rather than asserted by their authors. Where a premise remains unstated in this way, the reporting practices that would follow from stating it—specification of how components produce outcomes, verification that they were delivered as intended (49, 67), and corroboration of self-reported effects through independent sources—have no occasion to develop. The observation therefore concerns what these designs were built to answer, not what their investigators assume.

Mechanism underspecification is well documented even in established secular psychotherapies (16), and the broader field has responded: the Medical Research Council’s guidance for complex interventions progressively evolved toward requiring explicit mechanisms of impact, program theory, and process evaluation (19, 46). The reviewed programs have not yet fully incorporated these developments. A similar asymmetry is evident within the psychology of religion, where religious coping is extensively theorized as a process of change (68) yet rarely translated into explicit and testable intervention pathways. The absence of attention-matched control conditions further compounds this problem. Without such controls, intervention effects cannot readily be distinguished from non-specific influences such as attention, expectancy, or therapeutic contact, all of which have been shown to contribute substantially to observed outcomes (50, 51, 69).

Measurement structure carries comparable weight. Age-related changes in working memory, attentional control, and language comprehension can shape the self-report process itself (54), and data drawn from a single respondent under uniform response conditions remain vulnerable to common-method variance (53). The consequences are documented: moderate cognitive impairment among nursing home residents raises item non-response from 3.98 to 11.85% (70), and attentional difficulties introduce systematic bias into scale-based data (71). Where the person administering measures also delivered the intervention, or holds an institutional position on which residents depend, social desirability pressures may further compound these effects (48, 72). Under these conditions, single-source self-report designs without standardized cognitive screening leave the reliability of reported change difficult to evaluate independently of the delivery context. Because the present corpus is confined to a single religious tradition, it cannot establish whether this design profile is specific to Islamic-tradition interventions or characteristic of faith-based interventions more broadly.

Because the identified limitations operate in combination, piecemeal adjustment is insufficient: adding fidelity monitoring to a program without a specified mechanism, or new instruments without direct construct targeting, leaves the underlying problem intact.

Its contribution lies not in introducing entirely new psychological theories, but in integrating established developmental, existential, educational, and implementation-science perspectives into a single mechanism-based architecture organized around three psychosocial vulnerabilities that are not designated as primary outcomes in the reviewed literature. Erikson’s account of generativity and ego integrity provides the developmental logic of the framework, while Frankl’s conception of meaning-making supplies the interpretive rationale through which suffering, loss, and mortality become psychologically manageable. Realist evaluation and contemporary complex-intervention theory provide the methodological grammar through which these processes are rendered observable, attributable, and potentially testable.

Within this structure, the moral exemplarity of the Prophet Muhammad functions as a pedagogical organizing principle rather than as a mechanism in itself. This distinction is theoretically important. Prophetic narratives constitute the content through which participants engage the intervention, whereas the proposed mechanisms operate at the level of meaning-focused reappraisal, generativity reactivation, and cognitive reappraisal of mortality, as specified per module in Table 1. Consequently, the framework does not assume that intervention effects derive automatically from exposure to religious material. Rather, it proposes that culturally resonant narrative resources may facilitate specific psychosocial processes whose operation remains open to empirical examination.

This distinction also clarifies the framework’s relationship to broader psychological theory. The model does not assume theoretical equivalence between Islamic understandings of meaning and existential or humanistic approaches. Frankl’s account of meaning is ultimately grounded in individual discovery, whereas Islamic traditions locate meaning within alignment to divine purpose. The framework therefore operates at the level of functional convergence rather than ontological equivalence.

As a Hypothesis and Theory contribution, the scientific value of the framework rests not on demonstrated effectiveness but on its capacity to generate explicit and falsifiable propositions. Its central assumptions, from the proposed developmental sequencing to the mediating role of the specified mechanisms and the mechanism-specific rather than devotional action of Sīra-based content, remain open to empirical challenge, and Table 4 states each proposition with its falsification condition.

**Table 4:** Hypothesis audit: testable propositions, rival explanations, and falsification conditions.

Proposition	Testable prediction	Principal rival explanation	Falsification condition
P1 Mechanism attribution	Outcome change in each module is mediated by its specified mechanism (meaning-focused reappraisal; generativity reactivation; cognitive reappraisal/ego-integrity facilitation).	Change is produced by non-specific factors (attention, expectancy, relational contact).	Under an attention-matched active control, the mechanism indicators fail to mediate outcome change.
P2 Developmental sequencing	Module 3 gains are contingent on prior Module 1 and Module 2 gains.	Modules are independent; delivery order is immaterial.	A reordered or single-module delivery produces equivalent death-anxiety outcomes.
P3 Construct specificity	Direct targeting yields change on the dedicated instruments (UCLA, Rosenberg, Templer) beyond proximal constructs.	Effects are fully captured by proximal constructs (happiness, QoL, depression).	Proximal-construct change fully accounts for the observed effects on the three targets.
P4 Observance adaptivity	The psychosocial core produces comparable engagement across levels of religious observance.	Engagement requires active devotional commitment.	Engagement and outcome are contingent on devotional commitment (null effects among low-observance participants).
P5 Fidelity attribution	Under monitored fidelity, between-participant outcome variation tracks delivery variation.	Outcomes are unrelated to delivery quality.	Outcomes are invariant to documented fidelity differences.
P6 Reframing scope	Among participants who receive the solitude-reframing component, it reduces loneliness through change in the appraisal of aloneness, irrespective of whether placement was chosen or imposed.	The component operates only where the participant retains a sense of agency over placement, and is inert where placement was imposed.	Loneliness scores fail to improve among recipients reporting imposed placement while improving among the remainder.

Falsification logic follows mechanism-of-change standards (16); the rival-explanation column corresponds to the non-specific factors the recommended active-control and multi-source designs are constructed to adjudicate. Mediation testing of P1 presupposes completion of the indicator development sequence specified in Section 4.2.3; until that sequence is complete, P1 is testable through qualitative process evidence only.

**Table 5:** Comparative summary of reviewed programs and the design responses incorporated into the proposed framework.

Analytical problem area	Reviewed programs (*n* = 8)	Proposed framework
A1—Program theory articulation (3.1.1)		
CMO specification	Components described; mechanisms implicit; no testable CMO chains (“black box” interventions) across all 8 programs	Explicit CMO pathways per module component; mechanism indicators defined per session with candidate assessment procedures
Replicability infrastructure	Not addressed; procedural reporting varies across studies	TIDieR-aligned practitioner role specification; session delivery checklist; structured observation protocols
A2—Methodological integrity (3.1.2)		
Implementation fidelity	Not monitored in 7 of 8 programs; delivery, receipt, and enactment dimensions untracked	Four-level CIPP evaluation architecture; differentiated facilitator and outcome assessment roles; structured fidelity monitoring procedures
Control condition design	Passive or undescribed in all studies; no attention-matched equivalent; potential Hawthorne effects not addressed	Active control structurally matched in frequency, format, contact, and duration; narrative-matched version recommended for Islamic LTC settings, with a credible attention control as the stricter benchmark where locally acceptable
Role separation	Researcher delivered intervention and collected outcome data in 4 of 8 studies; detection bias risk	Facilitator, outcome assessor, and care staff observer roles explicitly differentiated
A3—Data source diversity (3.1.3)		
Data source architecture	Single-source self-report in 5 of 8; multiple scales in 2 studies (non-independent; same respondent, same conditions)	Multi-source mixed-method data architecture with embedded triangulation; pre-test, post-test, and follow-up assessment; MMSE ≥25 screening prior to inclusion
Cognitive capacity screening	Not assessed in 6 of 8 programs; standardized screening in 1 of 8 (MMSE); records-based ascertainment in 1.	MMSE ≥25 required for self-report outcomes; observational pathway for lower scores.
A4—Designation of core constructs as primary outcomes		
Primary outcome constructs	Happiness, QoL, depression, and general mental health designated as primary outcomes. Loneliness designated in 1 of 8 ((12), with a validated instrument); self-esteem in none, approached through the adjacent construct of self-acceptance in 1 of 8; death anxiety in none. No program designates more than one of the three.	Loneliness (UCLA Loneliness Scale), self-esteem (Rosenberg Self-Esteem Scale), and death anxiety (Templer DAS) as primary targets
Islamic theoretical grounding	Religious content as delivery medium; mechanism logic secular or unspecified; Sīra used narratively without pedagogical theorization	uswah ḥasanah tradition; ḥusn al-khātima and muḥāsabat al-nafs as module-specific conceptual anchors; hadith tarbawi framework
Modular sequencing	Single-program designs; no developmental ordering of vulnerability constructs	Three sequentially ordered modules with hypothesized developmental interdependence (loneliness → self-esteem → death anxiety)

QoL, quality of life; UCLA, UCLA Loneliness Scale; RSES, Rosenberg Self-Esteem Scale; DAS, Death Anxiety Scale; CMO, component–mechanism–outcome; CIPP, Context–Input–Process–Product; MMSE, Mini-Mental State Examination; LTC, long-term care; TIDieR, Template for Intervention Description and Replication. All design responses are theoretical propositions requiring systematic examination in future implementation studies.

At a methodological level, the study offers a specification template through which future Islamic-tradition interventions may be designed with explicit mechanisms, fidelity procedures, and evaluable program theory from the outset.

The mechanisms the framework mobilizes, namely meaning-focused reappraisal, generativity reactivation, and cognitive reappraisal, are established general mechanisms, not discoveries of this study. What has been absent from Islamic-tradition LTC research is their explicit specification, integration, and cultural instantiation within a single testable architecture. The contribution is therefore diagnostic and integrative: to our knowledge, it offers the first explicitly specified and falsifiable intervention architecture in this literature, rather than a claim of mechanistic novelty. That specification is complete at the level of component–mechanism–outcome pathways and incomplete at the level of mechanism measurement (4.2.3). The three claims the framework makes about these mechanisms are of different standing: that they are genuine change processes is established; that they can be activated through culturally resonant narrative content is supported indirectly, and by evidence drawn from outside institutional LTC (4.2.1); and that so activating them produces the proposed sequence of outcomes is the framework’s own hypothesis, stated for testing in Table 4.

The framework nevertheless remains conceptual: its mechanisms, developmental assumptions, and component–mechanism–outcome pathways await empirical validation, as detailed in the Limitations. Because it was derived from all retrieved studies meeting the predefined inclusion criteria rather than from a selective set of illustrative cases, the analysis offers a theoretically informed, though provisional, basis for future intervention development.

Ultimately, the present study advances a deliberately modest claim. It does not assert that prophetic-exemplar psychoeducation reduces loneliness, restores self-esteem, or alleviates death anxiety. Rather, it argues that such claims have not yet been formulated in a manner that permits rigorous mechanism testing, cumulative theory development, or explanatory evaluation. By identifying recurring explanatory limitations and translating them into an explicit mechanism-based framework, the study seeks to shift the field from the demonstration of outcomes toward the explanation of outcomes.

### 4.2 The proposed mechanism-based modular psychoeducational framework

Building on the analytical findings presented above, this section addresses RQ3 by developing a theoretically grounded, modular psychoeducational framework in which each design decision corresponds directly to one or more of the limitations identified in the Findings. Mechanism indicators are presented as candidate observational markers rather than validated instruments.

The framework comprises three sequential psychoeducational modules, detailed below. Module 1 targets loneliness through meaning-focused reappraisal and graded behavioral reinforcement; Module 2 targets self-esteem through generativity reactivation and the relational constitution of self-worth; and Module 3 targets death anxiety through cognitive reappraisal and structured mortality exposure. The foundations presented first are referenced forward to these modules throughout.

#### 4.2.1 Integrated theoretical and instructional design foundations

##### 4.2.1.1 Islamic theoretical grounding

The framework’s Islamic pedagogical foundation is the concept of uswah ḥasanah introduced above. It is not formalized as a single systematic pedagogical theory in the classical literature but is historically embedded in the practices of taʿlīm wa-tarbiya, within which the Sīra functions not merely as biographical narrative but as a resource for interpreting ethical and psychological orientations across the lifespan (20–22).

The Prophet’s engagement with older individuals, his normalization of death remembrance, and his affirmation of worth independent of functional capacity can thus be interpreted as pedagogically relevant patterns within this broader moral framework rather than as isolated historical anecdotes.

Empirical support for the mechanism plausibility of this adaptation is available. Cucchi (73) documents that behavioral activation can be grounded in prophetically inspired actions and that cognitive restructuring aligns with the ninth-century Muslim scholar al-Balkhī’s emphasis on restructuring cognitions within a religious framework. Qualitative evidence further indicates that Muslim participants explicitly prefer culturally resonant narrative content over secular equivalents (73). Among Muslim older adults, religiosity has been empirically associated with reduced loneliness, enhanced self-esteem, and improved life satisfaction through mechanisms including meaning-making and emotional regulation (74).

Two boundary conditions require acknowledgement. First, the framework’s universal ethical register must be distinguished from its theologically located grounding: the proposed mechanisms, including meaning-focused reappraisal, generativity reactivation, and cognitive reappraisal, are theorized to operate through relational and cognitive processes rather than exclusively through theological belief, with prophetic episodes functioning as culturally resonant narrative stimuli. Whether this distinction holds in practice requires process-level empirical examination. Second, the evidence base linking religiosity to psychological outcomes derives predominantly from community-dwelling rather than institutionalized older adults; the specific mechanisms through which Sīra-based content activates these processes in LTC settings remain an empirical question for future implementation studies.

##### 4.2.1.2 Philosophical foundations

The framework draws on three philosophically complementary traditions. Reconstructionism conceptualizes education as an intervention domain oriented toward restoring disrupted relational structures, a premise that maps directly onto LTC contexts in which institutional entry disrupts prior relational networks (75). Existentialist philosophy reframes loneliness and mortality not as pathological deficits but as meaning-oriented conditions (30, 37, 76). Within this existentialist register, Frankl’s (31) logotherapy extends the insight therapeutically, theorizing meaning-construction as the primary mechanism through which suffering becomes psychologically survivable. Humanistic educational thought provides the pedagogical register, emphasizing non-judgmental and supportive learning environments in which subjective experience is validated (77, 78).

These traditions converge on a shared premise: the vulnerabilities targeted reflect disruptions in meaning structures and relational bonds rather than isolated individual deficits. Sartre’s (76) formulation that existence precedes essence informs the framework’s positioning of participants as active meaning-makers rather than passive recipients of fixed identities, a premise directly relevant to the self-worth reconstruction work of Module 2.

##### 4.2.1.3 Developmental and psychological foundations

The framework’s mechanism logic draws on Erikson’s psychosocial theory as its primary developmental foundation. Jungian analytical psychology is incorporated as a supplementary interpretive lens, not as a source of empirically validated mechanisms, but as a descriptive vocabulary for identity-related disruptions associated with institutional transition. Erikson’s framework was selected over alternative lifespan theories [e.g., socioemotional selectivity theory; (79)] because its final two stages map directly onto the three vulnerability domains the framework targets: generativity versus stagnation onto self-structural vulnerability, and ego integrity versus despair onto existential vulnerability, with relational vulnerability addressed across both.

##### 4.2.1.4 Erikson’s psychosocial framework

Erikson’s (13) Theory of Psychosocial Development provides two mechanism-relevant propositions. The seventh stage, generativity versus stagnation, conceptualizes contribution beyond the self as a central developmental task. In LTC contexts, the loss of familial, vocational, and social roles disrupts the primary channels through which generativity is enacted. This produces stagnation characterized by purposelessness and erosion of self-worth. Empirical work supports this mechanism, linking generativity to wellbeing and its disruption to declines in self-worth (33). Module 2 directly targets this process.

The eighth stage, ego integrity versus despair, frames late-life development as the integration of one’s life into a coherent and meaningful whole. LTC entry compresses multiple developmental disruptions simultaneously through role loss, relational attenuation, and enforced passivity. This amplifies despair and reframes mortality as interruption rather than completion. Module 3 addresses this dynamic through mechanisms of meaning reconstruction and life coherence facilitation. Relational belonging, the focus of Module 1, is the precondition for both stages: the capacity for generativity and for ego integrity each presupposes a sustained sense of social connection, which institutional entry is the first to disrupt.

##### 4.2.1.5 Supplementary Jungian interpretive Lens

Jung’s contribution operates at a distinct interpretive level, supplying two sensitizing lenses. Jung’s concept of persona is used here as a heuristic descriptor for role-based identity organization (80). In LTC contexts, the removal of role structures may be interpreted through this lens as role-based identity disruption, consistent with the disorientation and perceived worthlessness often reported following institutional entry. The second lens concerns psychological avoidance surrounding mortality, the tendency to foreclose rather than engage with death-related material (80), which informs why Module 3 targets confrontation rather than symptom management.

##### 4.2.1.6 Educational design foundations

Andragogical principles ground learning in experiential and biographical knowledge (81–83), a premise enacted in the biographical sharing tasks employed across the modules. Transformative learning theory (84–86) identifies reflective dialogue and meaning reconstruction as the primary educational mechanisms through which existential challenges may be reinterpreted rather than merely managed, the instructional basis of Module 3’s meaning-reconstruction work. Social cognitive theory informs the behavioral activation dimension: low-threshold social and contribution tasks through which self-efficacy is hypothesized to be incrementally rebuilt via graded mastery and observational learning (87, 88), concentrated in Modules 1 and 2. At the instructional level, the ARCS motivation model (89) structures each session: attention is engaged through prophetic narrative stimuli; relevance is established through biographical connection; confidence is built through incremental contribution tasks; and satisfaction is consolidated through peer recognition and structured closure.

#### 4.2.2 Program logic and the component–mechanism–outcome Schema

The operational detail in the following module descriptions serves a specific analytical purpose: to make the proposed CMO pathways explicit enough for future studies to test, replicate, or falsify individual components. Session illustrations, task clusters, and mechanism indicators are operationalization targets rather than protocol mandates, and resource-constrained settings may adapt delivery format and task complexity without abandoning the underlying CMO logic.

The proposed sequencing, loneliness, self-esteem, death anxiety, reflects a theoretically motivated ordering. Two alternative orderings were weighed: sequencing self-esteem first, on the argument that social risk-taking requires a minimum self-worth threshold (6, 63), and sequencing death anxiety first, following Yalom’s (36) existential approach. The present order was selected on the grounds that relational belonging constitutes the most proximal and immediately addressable vulnerability upon institutional entry, providing the affective safety necessary for the more inward work of self-worth reconstruction and mortality engagement. Whether this ordering produces better outcomes than its alternatives is a primary question for future comparative studies. Within this order, Module 1 is hypothesized to establish the relational preconditions (connectedness and the experience of being recognized) for the work that follows; Module 2 to build the self-worth foundation that permits engagement with Module 3 without defensive avoidance; and Module 3 to draw on both foundations as enabling conditions for mortality engagement.

The framework operationalizes each session through a structured set of learning techniques drawn from adult education principles: biographical sharing, guided imagery, reflective writing, metaphor-based activities, and behavioral activation tasks. Differentiated instruction is incorporated throughout: enriched pathways for participants with higher functional capacity, and simplified, individually supported options for those with greater cognitive or communicative challenges, in recognition of the cognitive heterogeneity documented in the Findings. At the implementation level, the framework specifies not only session content but also the relational and environmental conditions under which mechanism activation is hypothesized to occur, including a non-judgmental, psychologically safe group climate and voluntary participation norms.

##### 4.2.2.1 Module-1: Belonging and social connectedness

*Primary outcome construct*: loneliness [UCLA Loneliness Scale; (7)]. Validated Turkish, Indonesian, and Persian adaptations are available and recommended for use in the respective cultural contexts (90–92).

*Islamic theoretical anchor*: The obligation the tradition places on the community toward its older members: respect for elders [(27), Adāb 66], their inclusion in consultation (28, 32), and the visiting of the isolated and unwell (ʿiyādat al-marīḍ). This is the module’s primary anchor because the condition it addresses is produced relationally, through the withdrawal of recognition; the reframing of solitude below operates within it.

*Cognitive reframing*: The Prophet’s retreats to the Cave of Ḥirāʾ and the practice of iʿtikāf are not offered as an analogue of the condition institutional residents occupy: that condition is involuntary and open-ended, whereas the prophetic retreats were chosen, time-bounded, and undertaken from a position of health. They serve instead as a culturally authoritative contrastive stimulus, in which being alone is not identical with being diminished, making available the distinction between solitude as a circumstance and loneliness as an appraisal of it (61). The reframing target is the appraisal, not the circumstance. A complementary episode, the Prophet’s practice of greeting every person encountered without distinction of status (ifshāʾ al-salām), extends this reframing in the opposite direction: even brief, low-threshold social contact is framed as an ethically meaningful act [(27), Adāb 142, (93), Adāb, 14].

*The proposed mechanism is meaning-focused reappraisal*: of solitude from threat to resource, and of social contact from obligation to meaningful engagement (30, 31). The proposed outcome pathway runs from reappraisal → reduced threat salience → increased tolerance for aloneness without loneliness. Candidate mechanism indicators: shifts in reframing language within session narratives; self-reported change in perceived control over solitude experience.

Boundary condition. Where solitude is experienced primarily as abandonment by family or community, this component is contraindicated in its standard form, since presenting chosen solitude as a model may increase rather than reduce distress. Intake screening identifies these participants, for whom the module proceeds directly to its behavioral-activation and relational-recognition components.

*Behavioral activation*: Episodes of social rejection at Ṭāʾif and enforced isolation during the Treaty of Ḥudaybiyyah supply the narrative framing for resilience-oriented behavioral engagement. The prophetic practice of encouraging visits to the sick and isolated (ʿiyādat al-marīḍ) functioned specifically to restore the social visibility of individuals who had become peripheral to community life [(42, 94), Birr, 12].

*Task clusters include*: Dyadic Listening and Role-Based Sharing, targeting perceived social support; low-threshold social contact initiation tasks, the Two Magnets task and brief visit activity, targeting the social withdrawal patterns documented in LTC loneliness research (3, 95); and Spiritual Reassurance Journals, targeting affect regulation and perceived relational safety reconstruction. The proposed mechanism is observational learning and graded behavioral reinforcement (87). Candidate mechanism indicators: frequency of self-initiated social contacts between sessions; facilitator-observed behavioral engagement with dyadic tasks.

*Biographical sharing and relational recognition*: Structured biographical sharing in small relational groups is designed to reactivate the relational dimension of belonging through social validation of self-narrative. The proposed outcome pathway runs from recognition experience → restored sense of social worth → enabling condition for Module 2 generativity work (13). Candidate mechanism indicators: participant use of recognition-acknowledgment language in session records; self-reported experience of being understood.

*Session illustration (Module 1, Session 3)*: Ṭāʾif account and ifshāʾ al-salām introduced; exclusion experiences named (10 min). Small-group reframing discussion; facilitator records reframing-language shifts (15 min). Two Magnets task assigned and journalled (20 min). One-sentence biographical sharing on feeling genuinely seen (10 min). Structured peer-acknowledgment close; recognition-language noted (5 min).

Candidate mechanism indicator items—Module 1 (one provisional item per specified component, completed by participants at the close of each session; 4-point Likert: 1 = not at all, 4 = very much). These items do not constitute a scale: they are not unidimensional, are not summed, and yield no total score. Each is a single-item marker of a distinct candidate construct, named in brackets after the component it belongs to. Their development is specified in Section 4.2.3.

1. [Cognitive reframing—reappraisal of solitude] During today’s session, I thought about my time alone differently than I usually do.
2. [Biographical sharing—perceived relational validation] I felt that other participants genuinely listened to what I shared.
3. [Behavioral activation—behavioral intention] I am willing to initiate contact with a peer before the next session.
4. [Relational recognition—perceived mattering] I felt that being here today mattered to someone other than myself.

Facilitator observation protocol (presence scale: 0 = absent, 1 = partial, 2 = present; one indicator per component): use of reframing language when discussing solitude; naming a specific peer for the Two Magnets task; unprompted contribution to the biographical sharing round.

*Theoretically expected proximal shifts:* increased social contact initiation frequency, biographical sharing engagement, and behavioral commitment adherence.

##### 4.2.2.2 Module-2: Dignity and recognition

*Primary outcome construct*: self-esteem [Rosenberg Self-Esteem Scale; (6)]. Validated Turkish and Persian adaptations are available (96, 97); for other target contexts, locally validated versions should be verified prior to use.

*Islamic theoretical anchor*: The prophetic principle that worth is constituted through intention and contribution rather than functional capacity, enacted paradigmatically in the episodes of Salmān al-Fārisī, Ibn Umm Maktūm, and Abū Quḥāfa.

*Recognition and contribution:* The Khandaq episode enacts the principle that accumulated knowledge and experience, irrespective of current social position, constitute genuine and valued resources (56, 58). The My Valuable Season reflections and Contribution Legacy Task draw on this logic. The proposed mechanism is generativity reactivation (13, pp. 61–65): restoring the contributory role whose disruption is the primary developmental source of self-worth erosion in LTC (33). Proposed pathway: restored contribution experience → reactivated generative orientation → self-worth reconstruction.

Candidate mechanism indicators: shifts in self-attribution language from external to internal locus; care staff records of between-session contribution behaviors.

*Proactive honoring*: The Abū Quḥāfa episode [(57), Vol. 4] informs structured peer-recognition rituals. The proposed mechanism is the relational constitution of self-worth (98). The prophetic injunction that ‘one who does not show respect to our elders is not of us’ [(27), Adāb 66] transforms peer recognition from interpersonal gesture into culturally authoritative obligation, strengthening mechanism activation through normative channels. Candidate mechanism indicators: reciprocal recognition exchange frequency; participant-reported experience of being valued.

*Unconditional worth*: The Ibn Umm Maktūm episode structurally decouples role-holding capacity from functional limitation [(57), Vol. 4]. The proposed mechanism is self-compassion activation through the decoupling of worth from performance criteria (99). This interpretive lens helps explain why relational recognition alone may not fully address self-worth erosion in LTC contexts: role loss may disrupt the social foundations on which conditional self-worth depends, rendering relational gains vulnerable to further functional decline. Self-compassion activation is theorized as the proximal shift that re-anchors self-worth in criteria that functional decline cannot undermine. Candidate mechanism indicators: increased self-compassionate language in written reflections; reduced self-critical attribution in contribution task debriefs.

*Session illustration (Module 2, Session 5)*: Khandaq and Ibn Umm Maktūm episodes presented; one retained skill, knowledge, or quality named (10 min). Life-wisdom sharing in triads; Recognition Cards exchanged, each naming one observed quality (20 min). Self-Compassion Reflection writing: “One thing I can still offer that has nothing to do with what I can physically do is…” (15 min). Structured peer-appreciation close; facilitator records self-compassionate language and locus of attribution (10 min).

Candidate mechanism indicator items—Module 2 (one provisional item per specified component, completed by participants at the close of each session; 4-point Likert: 1 = not at all, 4 = very much). These items do not constitute a scale: they are not unidimensional, are not summed, and yield no total score. Each is a single-item marker of a distinct candidate construct, named in brackets after the component it belongs to. Their development is specified in Section 4.2.3.

1. [Recognition and contribution—perceived retained contributory value] Today I shared something from my life that I feel still has value.
2. [Proactive honoring—perceived recognition by the group] I felt that my contribution was genuinely appreciated by the group.
3. [Unconditional worth—decoupling of worth from functional capacity] I noticed that my worth as a person does not depend on what I can physically do.
4. [Unconditional worth—relational form] I felt recognized today for who I am, not just for what I can do.

Facilitator observation protocol (presence scale: 0 = absent, 1 = partial, 2 = present; one indicator per component, complemented by a care-staff record): internal-locus attribution of contributions to personal knowledge or character; participation in reciprocal recognition exchange; self-compassionate rather than self-deprecating language; care-staff record of contribution behaviors initiated between sessions.

*Theoretically expected proximal shifts*: increased self-compassionate expression, competence attribution to personal effort, and sustained contribution task engagement.

##### 4.2.2.3 Module-3: Death awareness and preparation

*Primary outcome construct*: death anxiety [Templer Death Anxiety Scale; (8); complemented by qualitative narrative analysis]. Validated Turkish and Persian adaptations are available (100, 101); for other target contexts, locally validated versions should be verified prior to use.

*Islamic theoretical anchor*: The prophetic normalization of death remembrance as a meaning-generative orientation, and the concept of ḥusn al-khātima as a culturally grounded framework for mortality engagement. Within the Islamic framework, ḥusn al-khātima shares functional properties with Erikson’s ego integrity while grounding them in a theologically distinct ontology: meaning is disclosed through alignment with divine will rather than individually constructed. The framework operates at the level of this functional convergence without claiming ontological equivalence.

*Cognitive reappraisal*: The Prophet’s exhortation to frequent death remembrance [(34), Zuhd 3] and the hadith “The world is like the shade of a tree under which a traveller rests, then departs” [(102), Zuhd, 44] are engaged as reframing resources. The proposed mechanism is cognitive reappraisal of mortality from threat to meaning-dimension (31, 36). It is informed by Heidegger’s (37) account of authentic Being-toward-death as a structural resource rather than a threat to be managed, and operates at the functional level of conscious engagement with finitude, not at the ontological level of either existentialist or Islamic meaning-grounding. Candidate mechanism indicators: shifts in death-related language from threat-framing to acceptance-framing; reduced avoidance episodes in death-topic discussions.

*Meaning-making*: The Farewell Sermon’s prioritization of relational significance over material legacy informs the My Legacy Statement exercise and Life Coherence Review. The Life Coherence Review draws on muḥāsabat al-nafs, the Islamic practice of reflective self-examination, reframed here not as moral self-reckoning but as a structured life review through which participants take stock of the relationships and commitments that have given their life coherence. The prophetic emphasis on relational closure before death [(29), Wasāyā, 1; (103)] resonates with ḥusn al-khātima and the Eriksonian mechanism of ego integrity. The proposed mechanism is ego integrity facilitation (13, pp. 61–65).

*Proposed pathway*: coherence narrative construction → ego integrity → death acceptance as natural completion. Candidate mechanism indicators: narrative coherence markers in Legacy Statements; evidence of self-reflective stock-taking (muḥāsaba) in the Life Coherence Review; self-reported sense of life meaningfulness.

*Functional coping*: The prophetic practice of speaking directly with dying individuals about the nearness of death [(93), Janāʾiz, 1; (104)] enacts the guided mortality confrontation this component theorizes as necessary for durable change. The proposed mechanism is structured mortality exposure through meaning-anchored confrontation, reducing avoidance-based anxiety by integrating mortality into a coherent personal narrative rather than managing surface symptoms (36, 37). It thereby addresses the condition that symptom-management approaches leave intact, namely the avoidance orientation itself, by targeting that orientation directly through narrative engagement. Whether narrative engagement produces genuine integration rather than conscious acknowledgment alone is a primary empirical question for future implementation studies. Candidate mechanism indicators: reduction in topic-avoidance episodes across sessions; increased preparatory and reconciliation-oriented language in written reflections.

*Session illustration (Module 3, Session 8):* Farewell Sermon passage and the traveller’s shade hadith presented; one thing that has mattered most across life named (10 min). Guided imagery addressing a loved one, written into Legacy Statements (15 min). One unresolved relationship or unspoken appreciation identified; closure intention drafted (15 min). One-sentence sharing, each explicitly acknowledged by the facilitator (10 min). Legacy Statements retained in portfolios; avoidance-reduction and preparatory language noted (5 min).

Candidate mechanism indicator items—Module 3 (one provisional item per specified component, completed by participants at the close of each session; 4-point Likert: 1 = not at all, 4 = very much). These items do not constitute a scale: they are not unidimensional, are not summed, and yield no total score. Each is a single-item marker of a distinct candidate construct, named in brackets after the component it belongs to. Their development is specified in Section 4.2.3.

1. [Cognitive reappraisal—tolerance of mortality-related content] I was able to think about death today without feeling overwhelmed.
2. [Meaning-making—ego-integrity marker] I feel that my life has had meaning and value.
3. [Functional coping—preparatory orientation] I thought of something I would like to say or do before I die.
4. [Cognitive reappraisal—change in threat appraisal] Thinking about death today felt less threatening than it usually does.

*Facilitator observation protocol*: (presence scale: 0 = absent, 1 = partial, 2 = present; one indicator per component): use of acceptance- rather than threat-framing language in death-related discussion; production of a coherent Legacy Statement including at least one relational closure intention; engagement in death-topic discussion without avoidance behaviors. Legacy Statements are additionally assessed for thematic integration and closure language by a coder blind to quantitative outcome data.

*Theoretically expected proximal shifts*: increased capacity to engage death-related topics without avoidance, improved affective regulation, and increased use of preparatory and reconciliation-oriented language.

Boundary conditions: mortality-engagement components are contraindicated, or require individual-format adaptation, for participants with recent bereavement, a current terminal diagnosis, or a trauma-associated death history; intake screening for these conditions and the corresponding distress-management procedures are specified in the evaluation architecture (Section 4.2.3).

#### 4.2.3 Evaluation architecture: the CIPP model

The framework responds to the role-conflation limitation identified in the Findings through a multi-disciplinary facilitator structure. Each module is delivered by a domain specialist matched to its primary mechanism: relational and psychosocial processes by specialists in social work and psychology of religion, and Islamic historical and narrative content by specialists in Islamic history and religious education. Content expertise, participant relationship management, and outcome assessment are thereby kept functionally distinct, addressing the performance- and detection-bias risks associated with role conflation in the reviewed programs (48). This structural safeguard complements the evaluation architecture described below.

A pattern common across the reviewed studies is that measurement occurs only at intervention endpoints, leaving contextual rationale, component structuring, and operative psychosocial mechanisms outside the scope of evaluation. The CIPP model [Context–Input–Process–Product; (105)] was selected over alternative frameworks because it alone evaluates contextual rationale, implementation process, and outcome concurrently.

The four CIPP levels jointly supply what endpoint-only designs structurally cannot. The Context level documents the institutional and cultural conditions under which mechanisms are hypothesized to operate. The Input level anchors intake screening and facilitator qualification. The framework requires the Mini-Mental State Examination (106), with the ≥25 threshold governing eligibility for self-report outcome measures rather than participation itself. Intake also records whether placement was chosen or imposed, and whether solitude is experienced primarily as abandonment: the first is the moderator specified in Proposition P6, the second the boundary condition governing the Module 1 reframing component. Residents scoring below it take part through simplified engagement pathways and are assessed through observational and care-staff indicators only, with self-report data excluded from primary analysis. The Process level constitutes the fidelity-monitoring layer and draws on three complementary sources. A facilitator-completed Session Delivery Checklist records implemented activities, protocol deviations, and engagement. A brief structured participant session card captures perceived relevance, emotional safety, and engagement. Weekly institutional records, contributed by care staff, document behavioral indicators specific to each module’s proximal outcome domain: social contact initiation (Module 1), spontaneous contribution (Module 2), and willingness to engage in mortality-related conversation (Module 3). The Product level captures outcome data for comparative analysis. Although CIPP originates in educational evaluation, this cross-disciplinary application is consistent with the framework’s psychoeducational character.

Quantitative and qualitative data streams are integrated within the CIPP framework through a concurrent embedded mixed-methods design, enabling triangulation of effect direction with mechanism evidence. Quantitative outcome measures, namely loneliness, self-esteem, and death anxiety, are administered at pre-test, post-test, and follow-up. Qualitative data are collected concurrently through facilitator field notes and structured anecdotal observation forms completed after each session (independently by the lead and co-facilitator, reducing single-observer bias) participant reflective journals, and focus group interviews at the end of each module. At the close of each session, participants complete a structured behavioral commitment card specifying one concrete intention related to the session theme, assessing the enactment dimension of fidelity (49). Following program completion, semi-structured individual interviews using scenario-based prompts specific to each vulnerability domain assess whether the hypothesized coping mechanisms have been internalized beyond session participation. For the mortality-engagement module, Process-level safeguarding is specified. Facilitators monitor for acute distress through a defined indicator set, and a stepped response protocol provides pause, individual support, and penalty-free session exit. Participants meeting the contraindication criteria specified in Module 3 are referred for individual psychosocial or psychiatric support, and adverse events are logged in the weekly institutional record. Component, role, and mechanism specification follows the TIDieR framework (107) to strengthen replicability, and alignment with CONSORT guidelines supports the comparability of future effectiveness evaluations (108).

The candidate mechanism indicator items require development before they can carry inferential weight, and the framework specifies that development rather than assuming it. Five consecutive stages are required, the first of which is carried out by the expert panel described below.

1. Content review and item expansion. The initial item pools are submitted to that panel and expanded to a minimum of four candidate items per construct, since latent dimensionality cannot be evaluated on a single item.
2. Cognitive pretesting. Cognitive debriefing is conducted with LTC residents to examine item comprehension, readability, and emotional safety under the age-related response conditions documented in Section 4.1 (54).
3. Structural and model-fit validation. Because the items are administered repeatedly within sessions, observations are nested within participants and the structure is examined accordingly: multilevel exploratory factor analysis on a calibration sample, followed by multilevel confirmatory factor analysis on an independent validation sample, tests whether the retained items form the multiple correlated latent factors predicted by the component–mechanism–outcome logic rather than collapsing into a single dimension, with internal consistency (McDonald’s ω) reported per factor at the within-person level. The expanded pools are a development instrument: the set carried into routine session administration is the reduced subset these analyses retain.
4. Responsiveness and convergent validity. Because these indicators are designed to register change rather than to remain stable, conventional test–retest stability is not an appropriate criterion, since a retest correlation cannot distinguish true change between administrations from measurement error. What is required instead is evidence that item-level responses move with session content, and that the within-person trajectory of an indicator across sessions is associated with pre-test to post-test change on the primary outcome its pathway specifies.
5. Measurement invariance testing. Multi-group confirmatory factor analysis establishes invariance across levels of religious observance, which Proposition P4 presupposes. This requires an instrument that indexes observance as practice rather than belief content and that is validated in the target language; identifying such an instrument is a prerequisite of the invariance test rather than an assumption of it.

Until these stages are completed, the proposed items function strictly as observational prompts supporting facilitator-level and qualitative inference only, and no mediation analysis of the kind Proposition P1 requires may be conducted using them.

Two further steps precede implementation. The first is an independent appraisal: a panel of 9–12 members drawn from clinical geropsychology, gerontological care, social work, psychology of religion, religious education, Islamic history, and adult education, and not including the authors, receives the framework specification together with Tables 1, 4–6, one session outline per module, the evaluation architecture, and the control-condition options, and rates the framework on the same four dimensions and three-level scheme applied to the reviewed corpus, with written justification for each rating below present. Ratings are collected in a two-round modified Delphi format: independently in a first round, with aggregated anonymized results returned for a second round, at which a rating is retained when at least three quarters of panelists concur, and dissenting positions are reported rather than aggregated away. The second step is a bounded co-design cycle in which residents, family members, and front-line care staff decide three parameters: session length, the balance of spoken and written tasks, and which narrative material is withdrawn where it proves burdensome. Every resulting change, and every decision not to change, is recorded in a log. A Module 1 feasibility test then follows. Progression criteria are fixed here rather than deferred: consent among invited eligible residents at or above 60%, delivery fidelity on the Session Delivery Checklist at or above 80%, completion of scheduled session content at or above 75%, and attrition among participants eligible for self-report at or below 20%. Meeting three or four of these supports progression to a controlled evaluation; meeting two returns the specification to the panel; meeting fewer than two reopens the program theory. Any serious intervention-related adverse event halts the test irrespective of the other criteria. This sequence is specified here but has not been conducted, and no effectiveness claim is made in advance of it.

**Table 6:** Self-audit of the proposed framework against the scheme applied to the reviewed corpus.

Analytical dimension	Rating	Basis under the coding rule
Program theory articulation	Present	An explicit component–mechanism–outcome statement is identifiable for every module (Table 1)
Mechanism specification	Present	A named psychosocial process is tied to a defined component in every module
Construct–measurement alignment	Present	All three core constructs are designated as primary outcomes and measured with validated instruments
Methodological integrity	Absent	None of the four sub-criteria has been satisfied: fidelity monitoring, attention-matched control, role separation, and data-source independence are specified but not executed

The four dimensions and three levels are those applied to the reviewed programs in Table 2. Table 5 records the design responses the framework specifies; this table records whether those responses meet the execution standard applied to the corpus, and specification without execution is coded absent, as it was in Table 2. Construct–measurement alignment is rated on the primary outcome instruments, which are already validated; the candidate mechanism indicators are not, and the development sequence they require is specified in 4.2.3. The audit is the authors’ own; no independent appraisal has been conducted.

#### 4.2.4 Control condition design

The proposed framework assumes implementation within a controlled research design, with a structurally equivalent active control matched in session frequency, group format, facilitator contact, and duration, but whose content does not theoretically activate the hypothesized mechanisms. Three implementation considerations follow. Facilitator qualifications for the control condition may differ from those required for the intervention, a potential confound to be addressed through equivalence training. In Islamic LTC institutions, the complete absence of religious narrative may be perceived as culturally anomalous, differentially affecting engagement and attrition. A more feasible alternative is a narrative-matched condition that draws on Islamic narrative without the CMO-specified mechanism components, for instance general Sīra storytelling without structured cognitive reframing tasks. Whether this adequately isolates mechanism-specific effects from narrative exposure effects requires pilot examination. The stricter methodological benchmark remains a credible attention control (69): general psychosocial health material without prophetic narrative structure, biographical meaning-making tasks, or existential reframing components, which most cleanly isolates framework-specific effects from therapeutic contact, group participation, and expectancy, and which underpins the falsification condition specified in Table 4. The narrative-matched condition trades part of this isolation for cultural acceptability; where an attention control is locally acceptable, it remains the preferred comparator, with the choice justified in the study protocol. Given the vulnerability of the target population, a waitlist-crossover protocol is additionally recommended, whereby the control group receives the active comparator during the trial and the full intervention upon its completion.

#### 4.2.5 Applicability, observance adaptivity and transferability

The framework’s primary context of application is Islamic and Muslim-majority long-term care settings. The three-module structure is designed for 8 to 12 sessions of approximately 60 to 75 min, delivered in groups of 6 to 10 participants by a facilitator experienced in psychosocial care, counseling, or religious guidance. Outcome assessment is conducted by personnel independent of delivery, preserving role separation. The modular architecture permits phased delivery (for example, one module per month), reducing the implementation burden on resource-limited settings; whether this flexibility preserves the hypothesized developmental interdependence among modules is itself an implementation question for pilot study.

The three targeted vulnerabilities are not specific to any single tradition. Loneliness, diminished self-esteem, and death anxiety are shared features of institutional later life, and diverse traditions have long sought to render them meaningful, whether through Christian readings of suffering and mortality or through the secular meaning-centered and existential approaches of Frankl (31) and Yalom (36). The framework operates at this shared level. Within it, the moral exemplarity of the Prophet Muhammad occupies the pedagogical position that ritual or devotional practice occupies in many existing programs: prophetic episodes supply the narrative content, organized around universally recognizable moral qualities such as compassion, dignity, and patience rather than doctrinal material. The underlying processes are theorized to operate through relational and cognitive mechanisms rather than through the transmission of belief.

Applicability within a Muslim-majority population is heterogeneous, ranging from the highly observant to the culturally but not devotionally identified. The framework accommodates this range through a two-layered design: an observance-adaptive psychosocial core (belonging, self-worth, meaning-making, and cognitive reappraisal) that does not require active ritual compliance, and an adaptive periphery permitting the optional integration of devotional practice according to participant preference. The term observance-adaptive is preferred over belief-independent: the stronger claim that the mechanisms operate equivalently regardless of theological orientation is treated as an open empirical question; the more modest claim is that the core processes remain accessible across the spectrum of observance. Religious identification is thus positioned as a moderator of mechanism strength, not a precondition for its activation, a proposition the layered design renders empirically testable.

The architecture is therefore not in principle tradition-specific: the same component–mechanism–outcome structure could accommodate exemplar-based and meaning-centered content from other religious traditions, or from secular existential and logotherapeutic resources; what would change is the narrative content and its register, not the underlying mechanism logic. This separability extends the framework’s potential relevance to religiously plural and secular long-term care settings. Transfer beyond the Islamic setting is advanced, however, as a theoretical entailment of the content–mechanism distinction and a direction for future work, not as a validated extension. Table 4 renders the framework’s testability explicit, stating its six central propositions together with the principal rival explanation each must outperform and the condition under which it would be falsified.

### 4.3 Limitations

Several limitations warrant acknowledgement. First, the framework is conceptual and has not been empirically tested; its component–mechanism–outcome propositions require validation in the implementation studies outlined above. Second, the evidence base is a small, geographically concentrated corpus: eight programs from Iran, Türkiye, and Indonesia, drawn from peer-reviewed and graduate-thesis sources published between 2015 and 2025. The analysis is therefore interpretive rather than statistically generalizable, and the identified patterns may not extend beyond these contexts or to literature outside the search frame. Third, the framework is instantiated in Islamic content and its validated scope is confined to Islamic and Muslim-majority LTC settings; although its mechanism architecture is argued to be transferable in principle, such transfer would require tradition-specific adaptation and dedicated empirical examination and is not claimed here. In addition, because self-report eligibility is confined to MMSE ≥25, the framework’s primary evidence claims are restricted to cognitively intact residents. Fourth, the four-dimensional, three-level scheme applied to the reviewed corpus is applied to the framework itself in Table 6. Three dimensions meet the present threshold. Methodological integrity does not: its four sub-criteria are specified but unexecuted, and specification was not counted as satisfaction when the corpus was coded. The pedagogical content was appraised individually by seven specialists in curriculum and adult education, psychology of religion, religious education, and Islamic history and revised in the light of their comments, but that is expert consultation, not the stakeholder co-design and feasibility testing the 2021 MRC guidance places in the development phase (46). The appraisal, co-design, and feasibility sequence specified in Section 4.2.3 has not been conducted, and the audit in Table 6 is the authors’ own rather than an independent one. Fifth, the selection of Sīra and hadith content, although conducted through the procedure specified in the Method and constrained by authenticity screening and external appraisal, was theory-led: the search frame was derived from the vulnerability triad rather than from the sources themselves. A team working from the same source universe under different psychosocial constructs could arrive at a different set of episodes. This is a boundary of the content layer rather than of the mechanism architecture.

## 5 Future directions

These limitations define a research agenda rather than a terminus. Initial pilot studies of individual modules, beginning with the loneliness component, would offer the first test of the framework’s assumptions. Subsequent controlled trials incorporating all three modules could examine the hypothesized interactions among relational, self-esteem, and mortality-related pathways and, critically, whether change in the specified mechanisms mediates outcome change. Comparative studies across religious traditions and levels of cognitive functioning would then clarify the boundary between the framework’s portable architecture and its Islamic content. The most informative immediate step is not a full effectiveness trial but process-level qualitative work establishing whether the proposed mechanisms are activated as theorized and whether the observance-adaptive distinction holds in lived implementation, the two questions on which the framework’s empirical fate principally depends. If the proposed mechanisms receive empirical support, the lasting contribution of this work will not be a single intervention model but a reusable explanatory architecture through which spiritually grounded care can generate cumulative, transparent, and transferable knowledge.

## 6 Conclusion

This study set out from a structured diagnosis: across an indicative corpus of eight Islamic-tradition psychosocial interventions in long-term care, outcomes are reported while the explanatory infrastructure that would render them interpretable, namely named mechanisms, monitored delivery, balanced comparison conditions, and corroborated measurement, remains largely unspecified. Alongside this, and distinct from it, the vulnerabilities most prevalent in this population are not designated as primary outcomes in these programs’ outcome architectures. In response, it advanced what is, to our knowledge, the first explicitly specified and falsifiable intervention architecture in this literature. Three sequential modules grounded in the moral exemplarity of the Prophet Muhammad each link culturally resonant narrative content to named psychosocial mechanisms and candidate indicators, embedded in a four-level CIPP evaluation architecture with attention-matched control options. The architecture is proposed as transferable in principle beyond the Islamic context, an untested entailment for which independent validation in at least one non-Islamic setting is a precondition. The framework itself remains a set of hypotheses. Its value will be decided not by the plausibility of its assumptions but by whether future implementation studies confirm, refine, or falsify the pathways it specifies. If the field takes up this task, research on religiously grounded psychosocial care for older adults can move from demonstrating that culturally resonant interventions work to explaining how, for whom, and under what conditions they do.

## Data Availability

The original contributions presented in the study are included in the article/supplementary material, further inquiries can be directed to the corresponding author.
